# Overexpression of Adiponectin Receptor 1 Inhibits Brown and Beige Adipose Tissue Activity in Mice

**DOI:** 10.3390/ijms22020906

**Published:** 2021-01-18

**Authors:** Yu-Jen Chen, Chiao-Wei Lin, Yu-Ju Peng, Chao-Wei Huang, Yi-Shan Chien, Tzu-Hsuan Huang, Pei-Xin Liao, Wen-Yuan Yang, Mei-Hui Wang, Harry J. Mersmann, Shinn-Chih Wu, Tai-Yuan Chuang, Yuan-Yu Lin, Wen-Hung Kuo, Shih-Torng Ding

**Affiliations:** 1 Institute of Biotechnology, National Taiwan University, Taipei 10617, Taiwan; d04642002@ntu.edu.tw (C.-W.L.); scw01@ntu.edu.tw (S.-C.W.); 2 Department of Animal Science and Technology, National Taiwan University, Taipei 10617, Taiwan; d98626001@ntu.edu.tw (Y.-J.P.); d98626004@ntu.edu.tw (C.-W.H.); b02626004@ntu.edu.tw (Y.-S.C.); r04626025@ntu.edu.tw (T.-H.H.); r08626009@ntu.edu.tw (P.-X.L.); r09626024@ntu.edu.tw (W.-Y.Y.); mersmann@msn.com (H.J.M.); 3 Institute of Nuclear Energy Research, Taoyuan 325, Taiwan; d00642003@ntu.edu.tw; 4 Department of Athletics, National Taiwan University, Taipei 10617, Taiwan; hbeaverk@ntu.edu.tw; 5 Department of Surgery, National Taiwan University Hospital and College of Medicine, National Taiwan University, Taipei 10617, Taiwan

**Keywords:** adiponectin, adiponectin receptor 1, beige adipose tissue, brown adipose tissue, cold-induced thermogenesis, PET/CT scintigraphy, uncoupling protein 1 (UCP1)

## Abstract

Adult humans and mice possess significant classical brown adipose tissues (BAT) and, upon cold-induction, acquire brown-like adipocytes in certain depots of white adipose tissues (WAT), known as beige adipose tissues or WAT browning/beiging. Activating thermogenic classical BAT or WAT beiging to generate heat limits diet-induced obesity or type-2 diabetes in mice. Adiponectin is a beneficial adipokine resisting diabetes, and causing “healthy obese” by increasing WAT expansion to limit lipotoxicity in other metabolic tissues during high-fat feeding. However, the role of its receptors, especially adiponectin receptor 1 (AdipoR1), on cold-induced thermogenesis in vivo in BAT and in WAT beiging is still elusive. Here, we established a cold-induction procedure in transgenic mice over-expressing AdipoR1 and applied a live 3-D [^18^F] fluorodeoxyglucose-PET/CT (^18^F-FDG PET/CT) scanning to measure BAT activity by determining glucose uptake in cold-acclimated transgenic mice. Results showed that cold-acclimated mice over-expressing AdipoR1 had diminished cold-induced glucose uptake, enlarged adipocyte size in BAT and in browned WAT, and reduced surface BAT/body temperature in vivo. Furthermore, decreased gene expression, related to thermogenic *Ucp1*, BAT-specific markers, BAT-enriched mitochondrial markers, lipolysis and fatty acid oxidation, and increased expression of whitening genes in BAT or in browned subcutaneous inguinal WAT of AdipoR1 mice are congruent with results of PET/CT scanning and surface body temperature in vivo. Moreover, differentiated brown-like beige adipocytes isolated from pre-adipocytes in subcutaneous WAT of transgenic AdipoR1 mice also had similar effects of lowered expression of thermogenic *Ucp1*, BAT selective markers, and BAT mitochondrial markers. Therefore, this study combines in vitro and in vivo results with live 3-D scanning and reveals one of the many facets of the adiponectin receptors in regulating energy homeostasis, especially in the involvement of cold-induced thermogenesis.

## 1. Introduction

There are two distinct types of adipose tissues, brown (BAT) and white (WAT), in mammals including humans and mice. Contrary to the primary role of white adipocytes in lipid storage, brown adipocytes are responsible for increasing energy expenditure by maintaining thermogenesis [1]. Mice or rats constantly possess surgically obvious significant amounts of BAT, particularly around the interscapular region, even in adulthood. Given BAT weights relative to total body weights, mouse BAT is quite large. Previously, it is believed that, unlike mice, human BAT only exists in infants and recedes in the growing period [2]. Until 2009, metabolically active BAT was clearly mapped within the supraclavicular region, correlated with cold stimulation and inversely correlated with body max index (BMI), obesity, or aging [3,4,5]. Therefore, recent interests about BAT development and methods to activate human and mouse BAT reignite adipose researchers.

BAT is mainly responsible for non-shivering thermogenesis that increases energy expenditure by uncoupling adenosine triphosphate (ATP) generation in mitochondrial respiration and dissipating chemical energy in the form of heat to maintain core body temperature. The mechanism is primarily through thermogenic uncoupling protein 1 (UCP1). Moreover, this thermogenic process is even more pronounced under cold- or diet-induced thermogenesis [6,7]. 

Recently, under certain stimuli such as cold exposure [8], peroxisome proliferator-activated receptor γ (PPARγ) agonists [9] or β-adrenergic agonists [10], subcutaneous white adipose tissues also acquire brown-like adipocytes in white adipose depots with emerging UCP1-positive adipocytes, enhanced mitochondrial biogenesis and enriched BAT-specific mitochondrial markers. Processes of developing recruitable brown-like adipocytes in white adipose depots are termed beiging/browning of white adipocytes or simply beige/brite (brown-in-white) adipocytes [11]. Therefore, activating thermogenesis solely in classical BAT or browned WAT, or in both tissues together becomes a combined interest in obesity and diabetes research. 

Adiponectin, with molecular weights of 30 kDa, is one of the early identified adipocyte-secreted protein hormones (adipokines) circulating into the blood in mice [12,13]. Adiponectin belongs to the complement 1q (C1q) family with a C-terminal globular domain homologous to C1q [14]. Multimer complexes (bound through a collagen-like domain) of adiponectin, include trimer, hexamer and high-molecular-weight species are naturally formed and found in the circulation [15,16]. Receptors for adiponectin were first cloned as adiponectin receptors 1 and 2 (AdipoR1 and AdipoR2) from humans and mice [17]. We first cloned porcine counterparts corresponding to mouse/human AdipoR1 and AdipoR2 [18]. AdipoR1 and AdipoR2 each possesses seven transmembrane domains. However, structure, topology and function of AdipoR are distinct from common G-protein-coupled receptors [17]. AdipoR1 and AdipoR2 serve as main receptors of adiponectin for many metabolic functions in various tissues with AdipoR1 expressed universally, including in the skeletal muscle, liver, and adipose, and with AdipoR2 mostly expressed in the mouse liver [19].

Our previous studies generated transgenic AdipoR1 mice and found these mice defend against obesity, hepatosteatosis, and insulin resistance when fed a high-fat high-sucrose diet [20]. These AdipoR1-derived mouse mesenchymal stem cell transplantations ameliorate obesity-induced hepatosteatosis [21]. AdipoR1 also regulates mouse bone formation and osteoblast differentiation through the GSK-3β and β-catenin signaling [22]. These mice resist the decline of serum osteocalcin and GPRC6A expression in ovariectomized mice [23]. However, we also showed administering adiponectin or over-expressing AdipoR1 in mice enhances the inflammatory bowel disease [24]. Therefore, adiponectin and adiponectin receptors appear to play a complicated role and participate in an array of metabolic processes. 

The role of adiponectin on BAT or cold-stimulated thermogenesis in vivo is conflicted and related research about adiponectin receptors on BAT is limited. Here, the purpose of this study is to establish a cold challenge procedure for FVB mice and apply it to the transgenic mouse model over-expressing porcine AdipoR1. Unravel the role of AdipoR1 in the hypothermia condition to activate thermogenesis with cold-induced glucose uptake in vivo by live positron emission tomography/computed tomography (PET/CT) scanning. This was accompanied by in vitro analyses and in vivo supporting results such as histological examination of adipocyte size, surface body, and surface BAT depot temperature, thermographic imaging, and measurements of thermogenesis-related genes (thermogenic *Ucp1*, brown adipose specific markers, and brown adipose selected mitochondrial markers) in brown and browned/beiged white adipose tissues. 

## 2. Results

**Figure 1:**
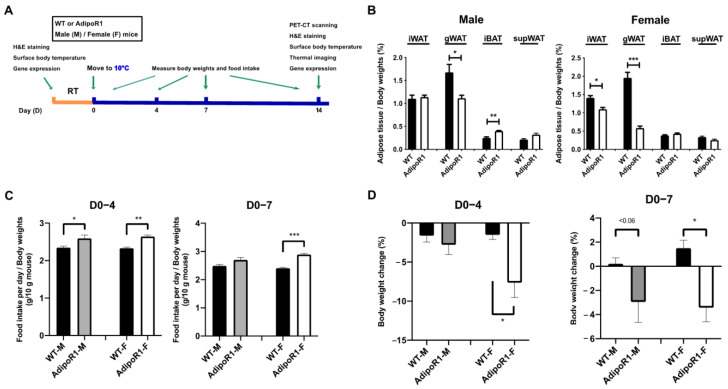
Body profiles of AdipoR1 mice upon cold exposure. (**A**) Wild-type (WT) and transgenic adiponectin receptor 1 (AdipoR1) mice of both sexes, male (M) and female (F), were housed in a cold-room (8–10 °C) for two weeks, i.e., 14 d. (**B**) Percentage of adipose tissue weights was expressed per unit body weights in WT or AdipoR1 mice. Subcutaneous inguinal white adipose tissues (iWAT), gonadal/epididymal white adipose tissues (gWAT), classical interscapular brown adipose tissues (BAT), and suprascapular white adipose tissues (supWAT) were harvested and weighted. (**C**) AdipoR1 mice had increased food intake (averaged g of feed per mouse per d/g of mouse body weights) while losing more body weight than WT mice. (**D**) AdipoR1 mice had significant body weight changes with enhanced body weight losses. Body weight changes were calculated and compared with each previous time point as percentage (%). Data were presented as means ± SEM (*n* = 3–8 for each group). * *p* ≤ 0.05; ** *p* ≤ 0.01; *** *p* ≤ 0.001.

After a pre-test confirming temperature and time length described in Materials and Methods, wild-type (WT) and transgenic porcine AdipoR1 mice were housed in a cold-room (10 °C) for two weeks. The workflow is shown in Figure 1A.

### 2.1. Enhanced Body-Weight Losses in AdipoR1 Mice during Cold Exposure

After cold induction, distinct white and brown adipose depots including subcutaneous inguinal white adipose tissues (iWAT), gonadal/epididymal white adipose tissues (gWAT), interscapular brown adipose tissues (BAT), and suprascapular white adipose tissues (supWAT) were collected. Transgenic AdipoR1 male mice had lower gWAT weights and greater iBAT weights compared with tissues in WT male mice. Transgenic AdipoR1 female mice had lower iWAT and gWAT weights compared with tissues in WT female mice (Figure 1B). These results showed different fat distribution under cold induction in WT or AdipoR1 mice in both sexes. 

Regarding food intake and body weight changes, overall, during the whole period of cold-induction, AdipoR1 male or female mice had greater accumulated food intake compared with WT mice, especially during periods of D0 to D4 and D0 to D7 (g per mouse per d / body weights) (Figure 1C). However, examination of body weight changes under cold environments indicated that both AdipoR1 male and female mice showed significant enormous body weight losses compared to WT mice, especially during periods of D0 to D4 and D0 to D7. WT female mice even gained body weights under cold exposure in D0-D7 (Figure 1D). These results showed that although transgenic AdipoR1 mice had increased food intake per unit body weight, AdipoR1 mice still had negative body weight changes during cold stimulation, indicating signs of cold-intolerance symptoms.

### 2.2. Diminished Cold-Induced Glucose Uptake In Vivo by PET/CT Scanning in Classical BAT and Browned SupWAT of AdipoR1 Mice

**Figure 2:**
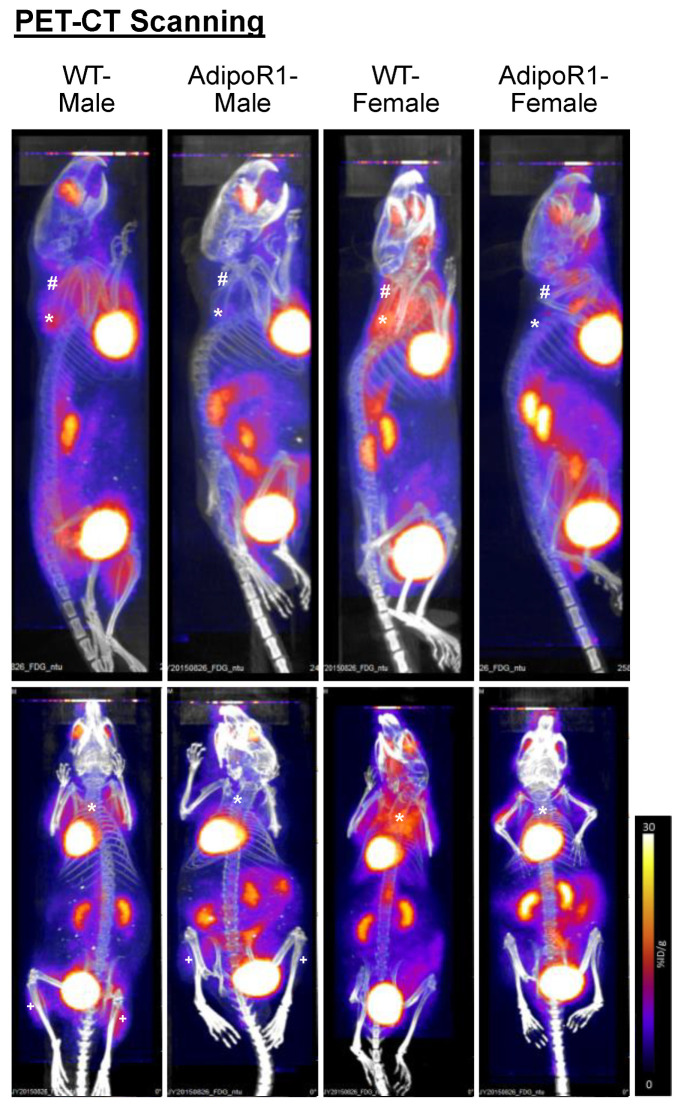
Inhibited cold-induced thermogenesis in vivo in classical brown and beige adipose depots of AdipoR1 mice. After two-week cold acclimation, WT and transgenic AdipoR1 mice were anesthetized and evaluated for cold-induced thermogenesis by live positron emission tomography/computed tomography (PET/CT) scanning. Timeline and procedures were described in Figure 1. Radiotracer of ^18^F-fluorodeoxyglucose (^18^F-FDG) was injected into the tail-vein to evaluate cold-enhanced glucose uptake in vivo. Asterisks (*) indicate classical iBAT and hashmarks (#) and plus signs (+) indicate supWAT and iWAT (potential depots for WAT browning or beiging), respectively. Procedures in different groups of mice were repeated at least twice. 3D-constructed videos are displayed in the Supplementary Materials.

To record live glucose uptake in vivo for BAT activity during cold stimulation in BAT and browned WAT, a PET/CT scanning with 2-deoxy-2-[^18^F]fluoro-D-glucose (^18^F-FDG) injection to the WT and transgenic AdipoR1 mice was applied after cold-stimulation. AdipoR1 mice showed significant reduction of cold-induced radio-labeled glucose uptake in vivo in classical iBAT, browned supWAT and browned iWAT compared to WT mice (Figure 2). Constructed 3D videos showed panoramic views of inhibited cold-induced glucose uptake in AdipoR1 mice (Supplementary Materials). Both male and female AdipoR1 mice showed strong diminution, especially pronounced in AdipoR1 female mice. These results demonstrated AdipoR1 mice clearly exhibit decreased cold-stimulated glucose uptake in BAT, browned supWAT, and browned iWAT.

### 2.3. Enlarged Adipocyte Size in Classical BAT and Browned iWAT of AdipoR1 Mice

**Figure 3:**
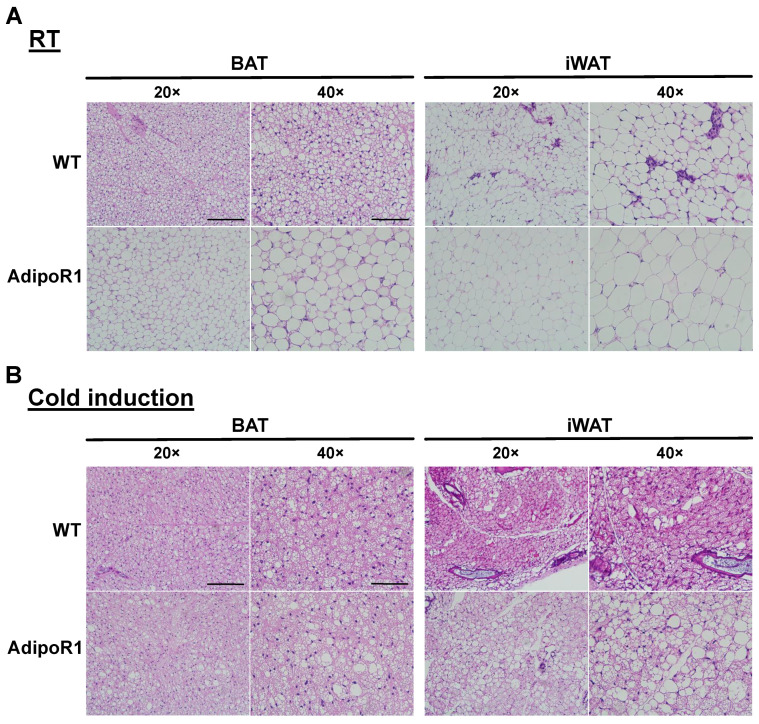
Increased adipocyte size in classical brown and beige adipose depots of AdipoR1 mice. Cold-acclimated WT and transgenic AdipoR1 mice were evaluated for the morphology of brown and beige adipose tissues. Timeline and procedures were described in Figure 1. Classical iBAT and iWAT (the potential white adipose depot for browning or beiging) in WT and AdipoR1 female mice (**A**) at room temperature or (**B**) in cold environments were harvested, fixed, sectioned, and evaluated for histology by hematoxylin and eosin (H&E) staining. Scale bar: 100 μm for 20×, 50 μm for 40× microscope.

To confirm the inhibited cold-induced thermogenesis by PET/CT scanning in AdipoR1 mice, the structure of BAT and browned iWAT (the primary WAT browning depot) were examined by hematoxylin and eosin (H&E) staining. Higher BAT activity was revealed in WT female mice versus WT male mice by PET/CT scanning (Figure 2). Therefore, we focus on comparing WT female mice with AdipoR1 female mice. AdipoR1 female mice both at room temperature (RT, Figure 3A) and under cold exposure (Figure 3B) showed increased adipocyte size with larger lipid droplets both in BAT and browned iWAT than WT female mice. These results indicated AdipoR1 mice acquire lower browning/beiging effects and maintain relative “whitening” phenotypes in classical BAT and browned iWAT than tissues in WT mice both at RT and under cold environments.

### 2.4. Decreased Surface Depot Temperature and Surface Body Temperature in Cold-Induced AdipoR1 Mice

**Figure 4:**
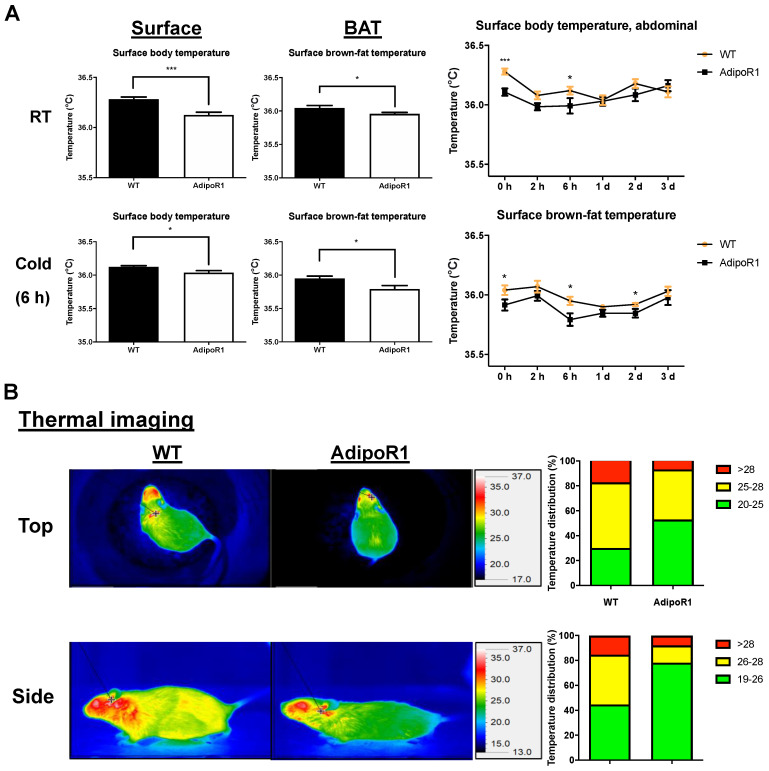
Reduced surface BAT depot temperature and surface body temperature of AdipoR1 mice. WT and transgenic AdipoR1 mice were evaluated for surface body temperature, (**A**) by infrared thermometer both at room temperature (RT) and after cold induction, and (**B**) by infrared camera after cold induction. Timeline and procedures were described in Figure 1. Measurements of surface body temperature (abdomen region) and surface depot temperature of BAT in (**A**) and infrared images from the thermographic surface temperature analysis in (**B**) in WT and AdipoR1 mice were both quantified and expressed as means ± SEM (*n* ≥ 8 for each group). * *p* ≤ 0.05; *** *p* ≤ 0.001.

To confirm the diminished cold-induced thermogenesis by PET/CT scanning in AdipoR1 mice, the surface brown adipose depot temperature and surface body temperature was examined by infrared thermometer and camera. AdipoR1 female mice under cold stimulation showed reduced surface BAT temperature and surface body temperature both before (RT) and after cold exposure (especially, 6 h) than WT mice (Figure 4A). Thermographic imaging also showed temperature distribution of surface body temperature in WT female mice was warmer than in AdipoR1 female mice (Figure 4B). These results, in line with PET/CT scanning and H&E staining, indicated AdipoR1 mice have reduced BAT and browned WAT activity.

### 2.5. Increased Adipose Expression of Adiponectin, Adiponectin Receptors, and Brown Adipocyte Markers in wt Female Mice Versus wt Male Mice under Cold Exposure

To evaluate expression of thermogenesis related genes in BAT and WAT beiging/browning for energy expenditure, multiple adipose tissues were assessed. White adipose tissues in certain depots possess the ability to be browned, termed beige adipocytes, according to the guide of an adipose tissue atlas [25]. Therefore, iBAT, supWAT, iWAT (two potential WAT browning depots), and gWAT (minimal potential for browning) were sampled.

**Figure 5:**
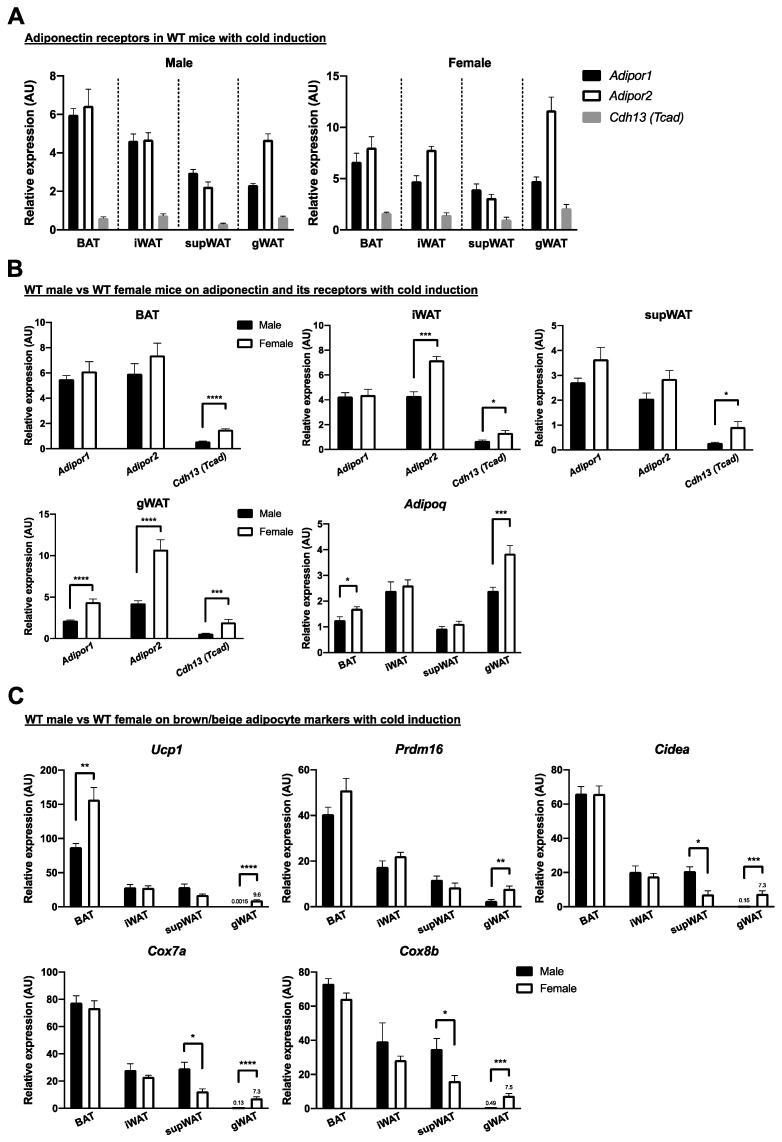
Female mice showed increased expression of adiponectin receptors and enhanced brown-adipocyte markers in classical brown and beige adipose depots after cold exposure. (**A**) Indicated adipose depots of male and female WT mice after cold acclimation were determined for expression of adiponectin receptors, including adiponectin receptor 1 (*Adipor1*), adiponectin receptor 2 (*Adipor2*), and T-cadherin (*Cdh13/Tcad*). (**B**) Comparison of expression of adiponectin receptors and adiponectin (*Adipoq*) between male and female WT mice was determined in iBAT, iWAT, supWAT, and gWAT, respectively. (**C**) Indicated adipose depots in male or female WT mice after cold acclimation were determined for expression of brown adipocyte (WAT browning/beiging) markers. Timeline and procedures were described in Figure 1. Classical iBAT, iWAT, supWAT (both of potential white adipose depots for browning or beiging) and gWAT in male and female WT mice were harvested, extracted and measured to evaluate adiponectin and its receptors and cold-induced thermogenic related genes (*n* = 3–8 for each group). Data were presented as relative means (normalized to β-actin in arbitrary units, AU) ± SEM. * *p* ≤ 0.05; **p ≤ 0.01; *** *p* ≤ 0.001; **** *p* ≤ 0.0001.

First, to uncover the role of adiponectin receptors and browning (beiging) effects between different sexes in cold environments, adiponectin, adiponectin receptors (AdipoR1, AdipoR2 and T-cadhrin), and brown adipocyte markers in BAT and in browned WAT were determined in WT male and female mice. Surprisingly, *Tcad* (mainly in hearts) was also detectable with Ct values of ~25 by qPCR detection. However, expression of *Adipor1* and *Adipor2* was still dominant in BAT, iWAT, supWAT, and gWAT under cold exposure compared to adipose *Tcad* expression (Figure 5A). In cold environments, WT female mice had greater *Adipor1* expression in gWAT, increased *Adipor2* expression in iWAT and in gWAT, enhanced *Tcad* expression in four adipose depots (BAT, iWAT, supWAT, and gWAT) and higher *Adipoq* expression in BAT and in gWAT compared with tissues of WT male mice (Figure 5B). Furthermore, WT female mice showed increased brown adipocyte markers, thermogenic *Ucp1* in BAT and in gWAT and *Prdm16*, *Cidea*, *Cox7a*, and *Cox8b* in gWAT (Figure 5C). These data indicated that WT female mice possess higher expression of adiponectin receptors and increased brown adipocyte markers than WT male mice in cold environments.

### 2.6. Reduced Adiponectin and Adiponectin Receptors in Adipose Tissues of AdipoR1 Female Mice Versus WT Female Mice under Cold Exposure

**Figure 6:**
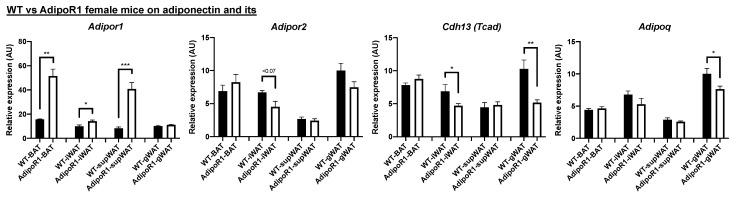
Increased expression of adiponectin receptor 1 and decreased expression of T-cadherin in classical brown or beige adipose depots of AdipoR1 mice after cold exposure. Indicated adipose depots of WT and AdipoR1 female mice after cold acclimation were determined for expression of adiponectin (*Adipoq*) and adiponectin receptors, including adiponectin receptor 1 (*Adipor1*), adiponectin receptor 2 (*Adipor2*), and T-cadherin (*Cdh13/Tcad*). Timeline and procedures were described in Figure 1. Classical iBAT, iWAT, supWAT (both of potential white adipose depots for browning or beiging), and gWAT in WT and AdipoR1 female mice were harvested, extracted, and measured to evaluate expression of adiponectin and its receptors (*n* = 3–8 for each group). Data were presented as relative means (normalized to β-actin in arbitrary units, AU) ± SEM. * *p* ≤ 0.05; ** *p* ≤ 0.01; *** *p* ≤ 0.001.

Given that WT female mice possess enhanced cold-induced glucose uptake in vivo by PET/CT scanning, and increased expression of adiponectin receptors and brown adipocyte markers than WT male mice in cold environments, we focused on comparing WT female mice with AdipoR1 female mice under cold exposure. Compared with WT female mice, AdipoR1 female mice showed increased expression of *Adipor1* in BAT, iWAT, and supWAT which confirmed that these transgenic mice were over-expressing adiponectin receptor 1 (Figure 6). Expression of *Adipor2* was no different in WT and AdipoR1 female mice. However, AdipoR1 female mice in cold environments showed reduced expression of *Tcad* in iWAT and gWAT and decreased *Adipoq* in gWAT (Figure 6). These results indicate these AdipoR1 female mice overexpressed adiponectin receptor 1 and somewhat had compensatory reduced expression of T-cadherin in certain adipose tissue depots.

### 2.7. Decreased Brown-Adipocyte Selective Markers in Classical BAT and Browned iWAT of AdipoR1 Female Mice under Cold Exposure

**Figure 7:**
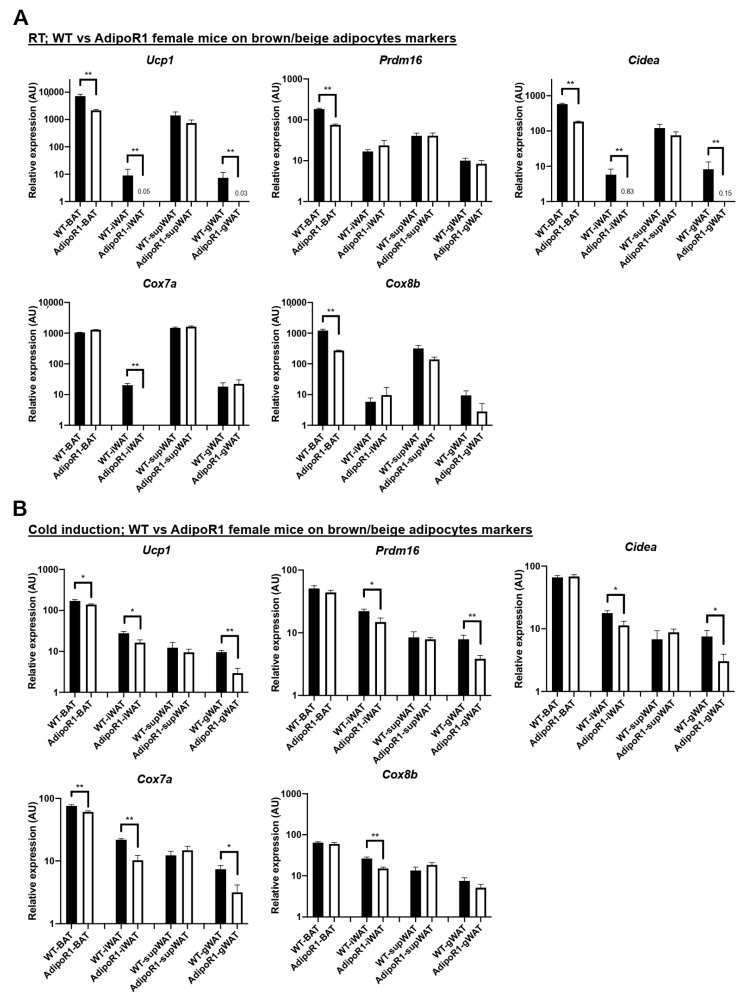
Reduced BAT thermogenic and mitochondrial markers in classical brown and beige adipose depots of AdipoR1 mice before and after cold-stimulation. Expression of brown adipocyte markers and BAT-enriched mitochondrial markers was evaluated in indicated adipose tissues of WT and AdipoR1 female mice, both (**A**) at room temperature (RT) before and (**B**) following the cold exposure at 10 °C. Timeline and procedures were described in Figure 1. Classical iBAT, iWAT, supWAT (both of potential white adipose depots for browning or beiging), and gWAT in WT and AdipoR1 female mice were harvested, extracted, and measured to evaluate cold-induced thermogenic related genes at RT or after cold acclimation (*n* = 3–8 for each group). Data were presented as relative means (normalized to β-actin in arbitrary units, AU) ± SEM. * *p* ≤ 0.05; ** *p* ≤ 0.01.

Compared with male mice, BAT activity by PET/CT scanning, expression of adiponectin receptors and expression of brown-adipocyte selective makers in BAT or in browned WAT were more pronounced in female mice. Therefore, we focused on classical BAT and subcutaneous iWAT in female mice for comparison of brown adipocyte markers in WT versus AdipoR1 mice. Brown adipocytes express highly distinct markers including thermogenic *Ucp1*, *Prdm16*, and *Cidea* and certain specific mitochondrial components *Cox7a* and *Cox8b* compared with white adipocytes. At RT (mild cold-induction), WT female mice already had greater expression of brown adipocyte markers, such as *Ucp1* in BAT, iWAT, and gWAT, than AdipoR1 female mice with other brown-adipocyte marker genes in similar patterns (Figure 7A). After prolonged exposure to cold to activate BAT and browned WAT (beiging), expression of thermogenic *Ucp1* in BAT, iWAT, and gWAT from AdipoR1 female mice was much lower compared to WT female mice. BAT-enriched mitochondrial marker *Cox7a* also had decreased expression in BAT, iWAT, and gWAT of AdipoR1 mice than in tissues of WT mice (Figure 7B). Other brown adipocyte markers *Prdm16* and *Cidea* and BAT-mitochondrial marker *Cox8b* in AdipoR1 female mice showed a resembling curtailed fashion. Therefore, these results showed that, both at RT and in response to prolonged cold-exposure, AdipoR1 female mice had reduced expression of thermogenic *Ucp1*, general brown adipocyte markers and BAT-enriched mitochondria markers in BAT and in browned WAT. 

### 2.8. Decreased Expression of Glucose Uptake, Lipolysis, and Fatty Acid Oxidation Genes in Classical BAT and Browned iWAT of AdipoR1 Mice under Cold Exposure

**Figure 8:**
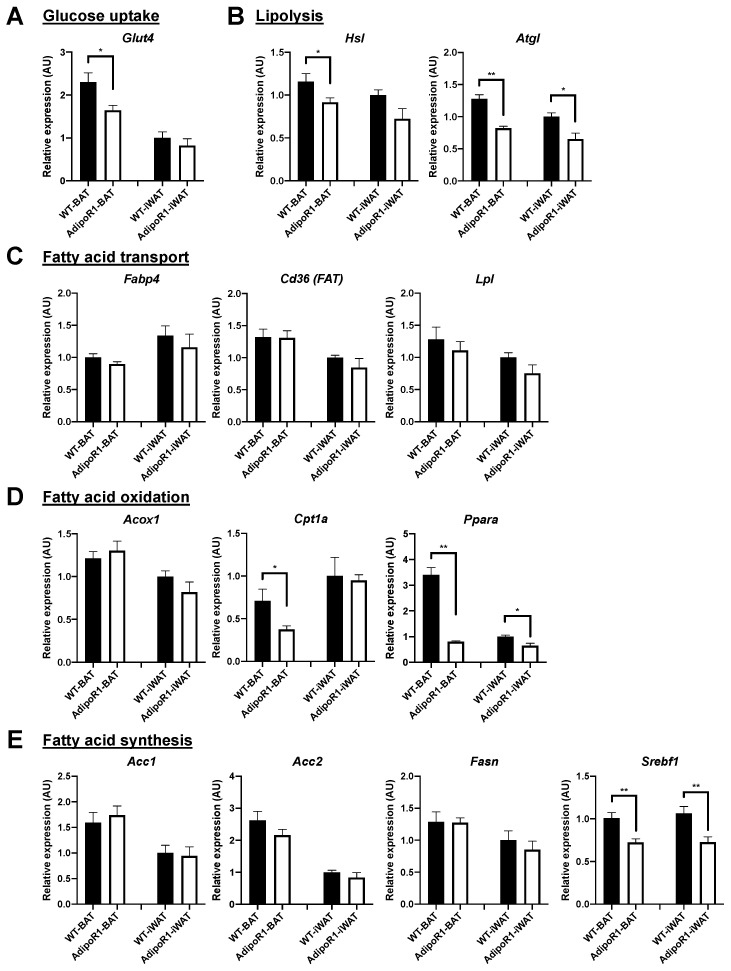
Reduced expression of glucose uptake, lipolysis, and fatty acid oxidation in classical brown and beige adipose depots of AdipoR1 mice after cold-stimulation. Gene expression related to (**A**) glucose uptake, (**B**) lipolysis, (**C**) fatty acid transport, (**D**) fatty acid oxidation, and (**E**) fatty acid synthesis was evaluated in indicated adipose tissues of WT and AdipoR1 female mice following cold exposure. Timeline and procedures were described in Figure 1. Classical iBAT and iWAT (the potential white adipose depot for browning or beiging) in WT and AdipoR1 female mice were harvested, extracted, and measured to evaluate adipose metabolism genes (*n* = 3–8 for each group). Data were presented as relative means (normalized to β-actin in arbitrary units, AU) ± SEM. * *p* ≤ 0.05; ** *p* ≤ 0.01.

To confirm inhibited cold-induced glucose uptake in vivo and reduced brown adipocyte markers in BAT and in browned iWAT of AdipoR1 female mice, gene regulation related to glucose uptake, to lipolysis, and to fatty acid transport, oxidation, and synthesis was determined. AdipoR1 female mice compared to WT female mice, upon cold exposure, had reduced expression of *Glut4* in BAT (related to glucose uptake), *Hsl* in BAT, *Atgl* in BAT and in iWAT (both related to lipolysis), *Cpt1a* in BAT, *Ppara* in BAT and in iWAT (both related to fatty acid oxidation), and *Srebpf1* in BAT and in iWAT (related to fatty acid synthesis) (Figure 8). However, expression of *Fabp4*, *Cd36*, *Lpl*, *Acox1*, *Acc1*, *Acc2*, or *Fasn* was not different between WT and AdipoR1 female mice (Figure 8). These results indicate AdipoR1 mice had decreased gene expression related to glucose uptake, lipolysis, and fatty acid oxidation that is congruent with in vivo results of lowered BAT activity of cold-induced glucose uptake and diminished brown adipocyte markers in AdipoR1 mice. 

### 2.9. Decreased Expression of Browning Genes and Enhanced Whitening Genes in Classical BAT and Browned iWAT of AdipoR1 Mice After Cold Exposure

**Figure 9:**
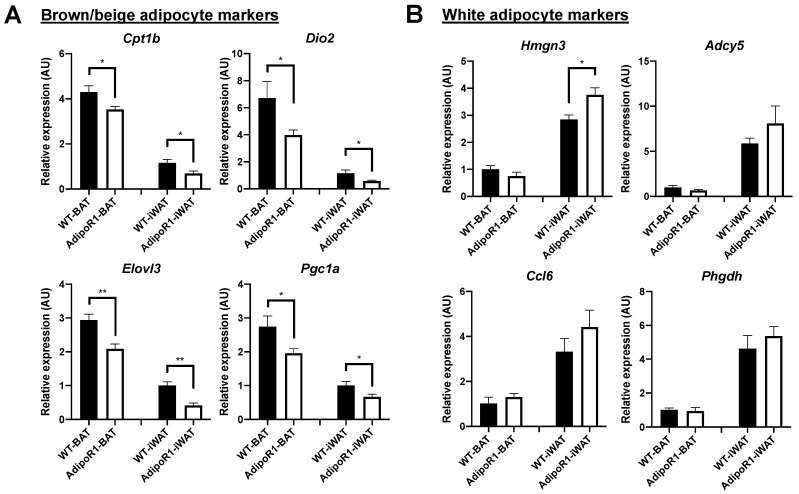
Reduced BAT markers (browning/beiging) and enhanced WAT markers (whitening) in classical brown and beige adipose depots of AdipoR1 mice after cold-stimulation. Expression of (**A**) brown and (**B**) white adipocyte markers was evaluated in indicated adipose tissues of WT and AdipoR1 female mice following cold exposure. Timeline and procedures were described in Figure 1. Classical iBAT and iWAT (the potential white adipose depot for browning or beiging), in WT and AdipoR1 female mice, were harvested, extracted, and measured to evaluate selective markers for brown or white adipocytes (*n* = 3–8 for each group). Data were presented as relative means (normalized to β-actin in arbitrary units, AU) ± SEM. * *p* ≤ 0.05; ** *p* ≤ 0.01.

Inhibited cold-induced glucose uptake in vivo, reduced brown adipocyte markers and enlarged adipocyte size revealed by H&E staining, in BAT and in browned WAT of AdipoR1 mice, all implicate diminished BAT function in BAT and browned WAT. To confirm this phenotype, additional gene expression [26,27] related to adipose browning (highly expressed in BAT vs WAT) and adipose whitening (highly expressed in WAT vs BAT) was selected and determined. AdipoR1 mice showed reduced expression of browning genes, *Cpt1b*, *Dio2*, *Elovl3*, and *Pgc1a* in BAT and in iWAT and increased whitening genes *Hmgn3* in iWAT than WT mice (Figure 9). These results indicate AdipoR1 mice maintain a relative adipose whitening phenotype in BAT and browned iWAT even after prolonged cold exposure.

### 2.10. Decreased Brown Adipocytes Markers in Differentiated Beige Adipocytes In Vitro Isolated from iWAT of AdipoR1 Mice

**Figure 10:**
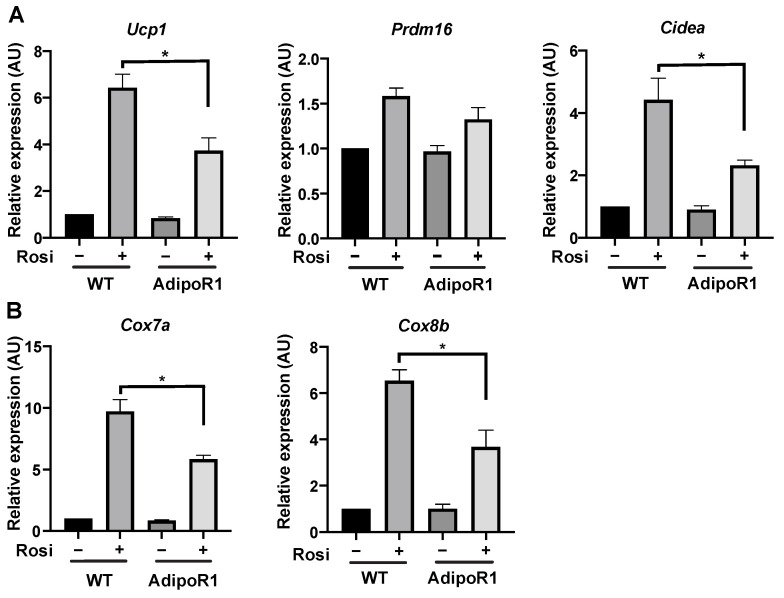
Decreased BAT thermogenic and mitochondrial markers in differentiated beige adipocytes from iWAT of AdipoR1 mice. Expression of (**A**) brown adipocyte markers and (**B**) BAT-enriched mitochondrial markers was evaluated by qPCR. Pre-adipocytes in the stromal/vascular fraction of subcutaneous iWAT (the potential depot for WAT browning or beiging) were derived from WT or transgenic AdipoR1 mice (*n* = 3) and differentiated into mature beige adipocytes with or without a synergistic browning agent of PPARγ, rosiglitazone (Rosi). Data were presented as relative means (normalized to β-actin in arbitrary units, AU) ± SEM. * *p* ≤ 0.05.

Subcutaneous inguinal WAT is the largest white adipose depot in mice for isolating pre-adipocytes and also meets with retaining great potentials for differentiating into either traditional white or beige (brown-like) adipocytes, especially under PPARγ-agonist stimulation during differentiation process [9]. To further confirm the in vivo results of abated cold-induced glucose uptake and inhibited expression of brown adipocyte markers in BAT and browned WAT, we isolated pre-adipocytes from iWAT and differentiated them into beige adipocytes with or without synergistic browning agents of PPARγ-agonists, rosiglitazone. Rosiglitazone clearly augments beige (browning of white) adipocytes with increased gene expression of brown adipocyte markers and brown-adipocyte mitochondrial markers (Figure 10). However, mature beige adipocytes differentiated with rosiglitazone from AdipoR1 mice showed reduced expression of brown adipocyte markers, thermogenic *Ucp1*, and *Cidea* compared with rosiglitazone-supplemented beige adipocytes from WT mice (Figure 10A). Brown-adipocyte enriched mitochondrial makers, *Cox7a* and *Cox8b*, showed similar effects of diminution in beige adipocytes from AdipoR1 compared to WT mice (Figure 10B). Therefore, these results showed both in vivo and in vitro results of reduced adipose expression of brown adipocyte markers in AdipoR1 mice. 

## 3. Discussion

Adiponectin is a beneficial adipokine defending against obesity-induced diabetes. However, the local effects of adiponectin on BAT activity or on cold-stimulated thermogenesis in vivo are in dispute. Investigation of adiponectin receptors on BAT is even more limited and not conclusive. The current research, to our knowledge, is the first to show that, in live in vivo cold-stimulated glucose uptake scanned by PET/CT scintigraphy, after prolonged cold-exposure for two weeks, mice over-expressing AdipoR1 had diminished ^18^F-FDG uptake and abated surface body temperature compared to WT mice. These suppressive results in AdipoR1 mice are supported by further analyses. Cold-acclimated AdipoR1 mice exhibited maintaining large lipid droplets of adipocyte size and reduced expression of brown adipocyte markers and of brown-adipocyte enriched mitochondrial markers, both in BAT and in browned WAT in vivo and in browned iWAT in vitro. 

### 3.1. PET/CT and Cold-Induced Glucose Uptake

PET/CT scintigraphy was first widely applied in clinical cancer research because cancer cells favor large amounts of glucose consumption. Therefore, radio-labeled glucose, ^18^F-FDG helps physicians spot glucose-consuming cancer cells in a tumor area [28,29,30,31]. Later, PET/CT scanning was adopted by endocrinologists searching for potential BAT in adult humans [3,4,5].

Nowadays, ^18^F-FDG PET/CT scanning is also used in preclinical mouse models for evaluating BAT activity or for brain research such as Alzheimer’s disease. For example, a recent research for adipose depot classification in the mouse adipose-tissue atlas used ^18^F-FDG or [^123/125^I]-β-methyl-p-iodophenyl-pentadecanoic acid coupled with PET/CT or SPECT/CT to image live cold-induced nutrient uptake in adipose tissues in vivo [25]. Depots of anatomically distinct brown, white, and beige (WAT browning) adipose tissues in mice are mapped using this method.

Based on the above mentioned mouse brown and white adipose-tissue atlas, in the current study, we choose (1) the interscapular region of brown adipose tissues (representing classical BAT), (2) the suprascapular and (3) the subcutaneous inguinal white adipose depots (both representing potential WAT browning sites), and (4) the gonadal region of white adipose tissues (representing dull WAT browning site) to evaluate cold-induced glucose uptake in BAT and in browning of WAT. By PET/CT scanning, our results showed inhibited cold-induced glucose uptake in BAT and browned WAT of AdipoR1 mice in both sexes.

Notably, this study also unveils that WT female mice possess greater amounts of cold-induced glucose uptake in vivo in BAT than WT male mice. Cold-acclimated WT female mice also had higher expression of thermogenic UCP1 in BAT and gWAT than WT male mice. These results are consistent with another study indicating that the β3-adrenergic receptor agonist CL316,243 induces enhanced brown adipocyte markers and mitochondrial respiratory chain proteins in gWAT of female mice than in gWAT of male mice, but without effects in iWAT [32]. Furthermore, estradiol, a major female sex hormone known to increase BAT activity, known to activate hypothalamic AMP-activated protein kinase (AMPK)-related [33] and augment bone morphogenetic protein 8b (BMP8b)-related thermogenic signaling [34], may account for the sex differences in BAT activity and consequently estradiol could elevate BAT activity in female mice.

Compared with WT male mice, cold-acclimated WT female mice also had elevated expression of adiponectin in BAT and gWAT, of AdipoR1 in gWAT, of AdipoR2 in iWAT and gWAT, and of T-cadherin in BAT, iWAT, supWAT, and gWAT. Female mice also had enhanced browning processes of cold-induced glucose uptake in BAT and thermogenic UCP1 in BAT and gWAT. Thus, our current study focused on female mice. Moreover, female mice with greater expression of adiponectin receptors and thermogenic UCP1 in adipose may be suitable for evaluating maximal effects of adiponectin receptor signaling on the cold-induced browning process in classical brown or browned white adipose tissues.

### 3.2. BAT and Cold-Induced Glucose Uptake

Without reducing food intake for cutting excessive supply of calories and impacting de novo lipogenesis, an alternative way to combat obesity-induced diabetes is increasing energy expenditure, potentially through activating brown adipose or browning/beiging of white adipose depots [35]. The thermogenic ability of BAT not only oxidizes fats but also consumes significant amounts of glucose as a metabolic “glucose sink”, especially activated by cold stimulation. For example, elevating BAT activity by cold exposure consumes significant amounts of glucose, which can be assessed by radio-labeled glucose coupled with PET/CT scanning, and also dramatically promotes clearance of plasma triglycerides and uptake into BAT through membrane receptor CD36 [36]. Therefore, activating BAT or WAT browning could be a viable way to defend against diet-induced obesity or type 2 diabetes.

In the clinically human translational application, β_3_-adrenergic receptor agonists, cold or other agents robustly activate human BAT and increase thermogenesis and energy expenditure both in healthy or obese human subjects. This cold stimulation in human studies can be achieved by staying in a cold-room or by putting on a running cooling water-filled suit with set temperature [37,38,39,40,41,42]. Therefore, application of PET/CT scanning in these human clinical studies or in above mentioned preclinical mouse models clearly showed that human and mouse BAT can be activated by cold-stimulation, thereby resulting in elevated energy expenditure and contributing to body fat reduction.

This current study also established a cold-induction procedure for mice to activate BAT or browned WAT assessed by PET/CT scanning. As opposed to a study of mice staying at 4 °C for C57BL/6 mice [43], we found it is much safer to perform cold-induction around 10 °C in a cold-room for our WT or transgenic AdipoR1 mice with FVB/NJNarl background. Therefore, strains of mouse species and transgene effects may account for different temperatures and periods of varying lengths for cold-induced thermogenesis.

### 3.3. Adiponectin/AdipoR1 on Modulating BAT and Browned WAT

Adiponectin is a beneficial adipokine to resist diabetes and causes a “healthy obese” state by increasing white adipose expansion and by limiting lipotoxicity in other tissues during high-fat feeding. For example, a classic mouse model over-expressing adiponectin in the prone-obese ob/ob background generates a super-obese phenotype with twice the size of the controls of already obese ob/ob mice. Nevertheless, these super-obese mice over-expressing adiponectin are protected from glucose intolerance and insulin resistance [44,45]. Therefore, these studies indicated that adiponectin reduces energy expenditure and increases energy preservation in white adipose tissues. Other studies also support that adiponectin knockout mice showed a lean phenotype or fat reduction [46,47,48].

However, exogenous administration of adiponectin to mice decreases plasma glucose, free fatty acids and triglycerides, increases muscle fatty acid oxidation and induces weight loss [49]. One of the functions of adiponectin is carried out through the activation of AMPK [46,50]. Therefore, short-term induction versus long-term supply of adiponectin and exogenous administration versus gene overexpression/deletion may cause discrepancy and result in inconsistent results of fat reduction or expansion.

In the current study, cold-acclimated AdipoR1 mice exhibited fat reduction of gWAT weights and showed whitening phenotype of iBAT assessed by H&E staining and by expression levels. AdipoR1 mice coupled with these fat alterations cannot resist acute cold exposure and flaunted lowered surface body and surface BAT depot temperature, accompanied with significant bodyweight losses. Therefore, these cold-acclimated AdipoR1 mice indicate implications of cold intolerance.

Non-shivering thermogenesis primarily occurs in brown adipose tissues. BAT is enriched with abundant mitochondria identified with many brown-specific mitochondrial makers, such as *Cox7a* and *Cox8b* [51]. Thermogenic UCP1 in mitochondria is responsible for uncoupled respiration from ATP synthesis and generation of heat to maintain core body temperature, especially during cold-induced hypothermia and in diet-induced thermogenesis [52].

In this study, we found that these AdipoR1 female mice, after prolonged cold exposure, showed not only inhibited cold-induced BAT activity of glucose uptake assessed by PET/CT but also concomitant strong reduction of brown adipocyte markers, including thermogenic gene *Ucp1*, brown adipocyte markers *Prdm16*, *Cidea*, *Dio2*, *Cpt1b*, *Elovl3*, and *Pgc1a*, and BAT-specific mitochondrial markers *Cox7a* and *Cox8b* in classical BAT or browned iWAT. Furthermore, AdipoR1 female mice at RT already showed this diminished pattern which is even more pronounced in cold environments. In vitro gene expression analysis of brown adipocyte markers in differentiated beige adipocytes from iWAT of AdipoR1 mice also support in vivo results of PET/CT scanning and expression of brown adipocyte markers.

Cold induction is known to decrease serum adiponectin concentrations through sympathetic activation in mice [53], especially evidenced in atherogenic conditions [54]. However, studies related to adiponectin receptors on cold-induced thermogenesis are limited. Our results of adiponectin receptor 1 on inhibition of thermogenesis are consistent with studies revealing that administering adiponectin also suppresses cold-induced thermogenesis and thermogenic UCP1 expression in mouse BAT [55]. Although the same study also showed that ablating adiponectin in mice elevates while abating AdipoR1 or AdipoR2 in mice decreases cold-induce thermogenesis in vivo [55]. These results are confounding and inconclusive. One possible speculation is that reduced thermogenesis by adiponectin may be simultaneously through multiple adiponectin receptors or adiponectin signaling. Individually ablating each adiponectin receptor (AdipoR1, AdipoR2, or T-cadherin) separately may actually enhance adiponectin signaling by causing compensatory increases in other adiponectin receptors. Therefore, both over-expressing (in our mouse model) or ablating individual adiponectin receptor could all augment adiponectin signaling, thereby diminishing cold-induced thermogenesis. However, detailed mechanisms await further investigation.

Cold-induced thermogenesis requires fatty acids as energy fuels, largely attributed to WAT lipolysis. In response to cold exposure under hypothermia, the sympathetic nervous system sends signals to activate lipolysis and thermogenesis. Circulating liberated fatty acids from WAT lipolysis are taken up by BAT for mitochondrial oxidation and thermogenesis [56]. Mechanistically, adiponectin suppresses basal and catecholamine-induced lipolysis in white adipocytes mediated by inhibited protein-kinase-A signaling [57]. Therefore, adiponectin decreases lipolysis in WAT which could result in decreased fuel sources for other tissues including BAT.

Recently, a new study also showed adiponectin or receptor agonist AdipoRon suppresses cold-induced thermogenic gene expression and energy expenditure in vivo through limiting immune-cell ILC2s-activated thermogenesis in BAT or beige (browned WAT) adipose tissues [58]. Therefore, this current study showed that reduction of cold-induced thermogenesis in mice over-expressing AdipoR1 is congruent with other studies of inhibited thermogenesis in mice administered adiponectin and of increased thermogenesis in mice with ablated adiponectin. Therefore, the combination of above-mentioned results of inhibited BAT thermogenesis by adiponectin or receptor overexpression might be partly through fatty acid retention in WAT with diminished lipolysis in WAT, resulting in decreased supply of fatty acids as thermogenic substrates. Moreover, we also found that over-expressing AdipoR1 suppresses WAT browning both in vivo and in vitro. Hence, in our mouse model, AdipoR1 inhibits cold-induced thermogenesis in both classical BAT and browned WAT.

To conclude, the live ^18^F-FDG PET/CT scanning for measurements of cold-induced BAT activity in mice showed that mice over-expressing adiponectin receptor 1 exhibit reduced cold-induced glucose uptake in BAT and browned supWAT, accompanied with decreased surface body and surface BAT depot temperature and increased adipocyte size of whitening morphology in BAT and browned iWAT. Further in vitro and in vivo analyses indicated expression of thermogenic gene *Ucp1*, brown fat-specific markers and brown adipocyte-enriched mitochondrial markers are largely attenuated in BAT or browned WAT of AdipoR1 mice. However, white adipocyte markers in BAT or browned WAT are maintained or elevated in AdipoR1 mice. These combined results with live PET/CT scanning reveal the role of adiponectin receptor 1 in regulating energy homeostasis, especially in cold-induced thermogenesis. Unveil detailed mechanisms underlying adiponectin signaling on brown adipose tissues or browning/beiging of white adipose tissues may provide insights for therapeutic application in obesity-associated metabolic diseases.

## 4. Materials and Methods

### 4.1. Animals and Diets

Transgenic FVB/NJNarl mice over-expressing porcine adiponectin receptor 1 were generated through the pronuclear microinjection of fertilized eggs, and bred as previously reported [23,24]. Briefly, founder mice were crossed with WT mice to generate F1 heterozygous (AdipoR1^+/−^) offspring. The F1 heterozygous mice were backcrossed with WT mice to generate the offsprings as F2 mice. F2 heterozygous mice were double-crossed to generate progeny of homozygous (AdipoR1^+/+^) littermates. These F3 littermates were checked as homozygous mice via quantitative real-time PCR analyses using DsRed2 primers (Supplementary Table S1) and GFP/RFP-fluorescent checking systems. All experiments were performed on homozygous offsprings starting from the F4 or later generations housing at the mouse facility in our department.

Adult age-paired male and female mice (>12 weeks old) of WT or with the transgene, from the F4 or later generations, were randomly assigned for each experimental group (*n* ≥ 3 for each sex per group), maintained at room temperature or at a cold-room with a light-dark cycle of 12: 12 h, and provided with feed (LabDiet 5001; LabDiet, St Louis, MO, USA) and water ad libitum. The animal experiments were approved by the Institutional Animal Care and Use Committee at National Taiwan University, Taipei and Institute of Nuclear Energy Research, Taoyuan, Taiwan (NTU-103-EL-42, NTU-106-EL-00088, NTU-108-EL-00076).

### 4.2. Cold-Acclimation Procedure

First, a pre-test of cold-induction protocol for our mouse strain was implemented to evaluate cold-stimulated thermogenesis in mice. Because different genetic inbred or outbred backgrounds of mice may respond very differently regarding cold tolerance at discrete low temperature and exposure times, a pre-trial with small numbers of WT and transgenic AdipoR1 mice (*n* = 3) in the FVB/NJNarl genetic background were housed in a cold-room at 4, 7, or 10 °C. Below 10 °C, WT or transgenic mice cannot tolerate the cold for more than 7 d. This was especially prevalent at 4 °C (data not shown). Therefore, in the following experiments, cold-induced thermogenesis in WT or AdipoR1 of FVB/NJNarl mice will be evaluated at about 10 °C.

Adult WT or AdipoR1 transgenic mice were divided and maintained on a regular chow diet. For cold exposure experiments after the pre-test, mice were transferred into a cold-room (8–10 °C) for two weeks and provided with feed and water ad libitum, with the same light-dark cycle of 12 h–12 h. Mice were closely monitored in cold environments and checked for their health and behavior at least once per day to prevent widespread sudden death from cold intolerance. Early termination will be initiated if extensive sudden death is observed.

### 4.3. Sample Collection and Preparation

Food intake and body weights of each group were measured on d 0, 4, 7 and 11. For tissue sampling, mice were sacrificed using carbon dioxide. Peripheral adipose tissues, including subcutaneous inguinal, gonadal, and suprascapular white adipose tissues, and interscapular brown adipose tissues, were harvested, weights recorded, snap-frozen in liquid nitrogen, and stored at −70 °C for future analysis.

### 4.4. PET/CT Scanning

After two weeks of cold acclimation with at least 6 h of terminal fasting, mice were escorted to the Institute of Nuclear Energy Research, Taoyuan, Taiwan for the ^18^F-FDG PET/CT Scanning (Mediso NanoPET/CT, Mediso Medical Imaging Systems, Budapest, Hungary). Before radiotracer injection, mice were anesthetized with 2% isoflurane injected through a tail vein. Then, 150 μCi of locally synthesized ^18^F-FDG was injected into the tail vein of the mice and mice were held in cages for 1 h waiting for biodistribution before scanning. Dynamic emission scans for each WT or AdipoR1 mouse were acquired for 20 min. The mice were constantly anesthetized with 0.1% isoflurane in air during the imaging. Results of ^18^F-FDG PET/CT images were coregistrated (constructed) using a PMOD software (PMOD Technologies LLC, Zurich, Switzerland) to estimate the radioactivity concentration. Volumes of interest and the activity were expressed as %ID/g.

### 4.5. Histological Analysis by H&E Stain

To evaluate the structure of interscapular BAT and subcutaneous iWAT, adipose depots were harvested, fixed in 10% formalin and then embedded in paraffin. The paraffin-embedded specimens were sliced at 4 μm using a microtome. The sections were stained with H&E to observe the pathological morphology of mouse adipose tissues.

### 4.6. Depot Surface Temperature Measurements and Thermographic Imaging

WT and transgenic AdipoR1 mice, before or after housing in the cold, were assessed for regional surface temperature on skins above brown adipose tissue areas and on the abdomen by an infrared thermometer (HFS-1000, Thermofinder Plus, HuBDIC, Gyeonggi-do, Korea). Readings were recorded with constant values at least 3 times.

Thermography was performed using an infrared camera (IRM-P384G, Ching Hsing Computer-Tech Ltd., Taipei, Taiwan) on WT and transgenic AdipoR1 mice acclimated at a cold room. No anesthesia was applied to avoid disturbing mouse body temperature with human-handling.

### 4.7. Gene Expression Analysis by qPCR

Total RNA was extracted from mouse tissues and cells by the TRIsure reagents (Bioline, Meridian Bioscience, London, UK), with contaminating genomic DNA later removed by the TURBO-DNase free kit (AM1907; Thermo Scientific, Waltham, MA, USA), followed by reverse transcription into cDNA using the High Capacity cDNA Reverse Transcription kit (Thermo Scientific). Quantitative real-time polymerase chain reaction (qPCR) was performed using a SensiFAST SYBR Hi-ROX Kit (Bioline) on an ABI StepOnePlus Real-Time PCR System (Applied Biosystems, Thermo Scientific) or a LightCycler 480 Instrument II (Roche Diagnostics, Indianapolis, IN, USA). qPCR was performed as follows; activate enzyme at 95 °C for 2 min, and then 40 cycles of 95 °C for 5 s and 60 °C for 30 s with a final extension of 60 °C for 1 min. Primers used for amplification of qPCR reaction are listed in Supplementary Table S1. The relative value for each target gene was normalized to β-actin expression in the same sample. The threshold cycle (Ct) values were obtained, and relative gene expression was calculated using the comparative Ct method. Amplification of specific transcripts was further confirmed by melting-curve analysis and agarose-gel electrophoresis to check resulting product specificity.

### 4.8. Cell culture for Isolation and Differentiation of Mouse Stromal/Vascular Cells into Mature Adipocytes

Mouse pre-adipocytes within the stromal/vascular fraction (SVF) from subcutaneous iWAT were isolated from adult WT or transgenic AdipoR1 mice. Detailed procedures for pre-adipocyte isolation, cell culture and differentiation into mature adipocytes were described previously with slight modification [59,60,61]. Briefly, the mouse subcutaneous inguinal region of WAT was surgically dissected, minced and digested with collagenase (C6885, Sigma-Aldrich, St. Louis, MO, USA) and then filtered through chiffon. Pre-adipocytes within SVF were derived through a series of centrifugation and washing steps with red-blood-cell-lysis before the final washing step. Then, pre-adipocytes were suspended and seeded on tissue-culture treated plates containing medium of Dulbecco’s Modified Eagle Medium/F12 (Gibco DMEM/F12, Thermo Scientific), 10% fetal bovine serum (Thermo Scientific) and 1% antibiotics (penicillin, streptomycin and amphotericin B; Biological Industries, Kibbutz Beit-Haemek, Israel). Cells were incubated at 37 °C in air containing 5% CO_2_ until confluency. Confluent pre-adipocytes were subsequently switched to induction medium with or without the PPARγ-agonist rosiglitazone for adipocyte differentiation. After around 7 to 9 d, mature adipocytes with at least 70% differentiation were collected for further analysis.

### 4.9. Statistical Analysis

Data were expressed as means ± standard error of the mean (SEM). Results of two groups were compared and determined for statistical significance by the Student’s *t*-test (Graphpad Prism 6; Graphpad Software, San Diego, CA, USA). The *p*-values ≤ 0.05 were considered statistically significant.

## Data Availability

The data that support the findings of this study are available from the corresponding author upon reasonable request.

## Supplementary Materials

Supplementary materials can be found at https://www.mdpi.com/1422-0067/22/2/906/s1.

## References

[1] Kusminski C.M., Bickel P.E., Scherer P.E. (2016). Targeting Adipose Tissue in the Treatment of Obesity-Associated Diabetes. Nat. Rev. Drug Discov..

[2] Bartelt A., Heeren J. (2014). Adipose Tissue Browning and Metabolic Health. Nat. Rev. Endocrinol..

[3] Cypess A.M., Lehman S., Williams G., Tal I., Rodman D., Goldfine A.B., Kuo F.C., Palmer E.L., Tseng Y.-H., Doria A. (2009). Identification and Importance of Brown Adipose Tissue in Adult Humans. N. Engl. J. Med..

[4] Van Marken Lichtenbelt W.D., Vanhommerig J.W., Smulders N.M., Drossaerts J.M.A.F.L., Kemerink G.J., Bouvy N.D., Schrauwen P., Teule G.J.J. (2009). Cold-Activated Brown Adipose Tissue in Healthy Men. N. Engl. J. Med..

[5] Virtanen K.A., Lidell M.E., Orava J., Heglind M., Westergren R., Niemi T., Taittonen M., Laine J., Savisto N.-J., Enerbäck S. (2009). Functional Brown Adipose Tissue in Healthy Adults. N. Engl. J. Med..

[6] Enerbäck S., Jacobsson A., Simpson E.M., Guerra C., Yamashita H., Harper M.-E., Kozak L.P. (1997). Mice Lacking Mitochondrial Uncoupling Protein Are Cold-Sensitive but Not Obese. Nature.

[7] Feldmann H.M., Golozoubova V., Cannon B., Nedergaard J. (2009). UCP1 Ablation Induces Obesity and Abolishes Diet-Induced Thermogenesis in Mice Exempt from Thermal Stress by Living at Thermoneutrality. Cell Metab..

[8] Qiang L., Wang L., Kon N., Zhao W., Lee S., Zhang Y., Rosenbaum M., Zhao Y., Gu W., Farmer S.R. (2012). Brown Remodeling of White Adipose Tissue by SirT1-Dependent Deacetylation of Pparγ. Cell.

[9] Ohno H., Shinoda K., Spiegelman B.M., Kajimura S. (2012). PPARγ Agonists Induce a White-to-Brown Fat Conversion through Stabilization of PRDM16 Protein. Cell Metab..

[10] Wang Q.A., Tao C., Gupta R.K., Scherer P.E. (2013). Tracking Adipogenesis during White Adipose Tissue Development, Expansion and Regeneration. Nat. Med..

[11] Wu J., Boström P., Sparks L.M., Ye L., Choi J.H., Giang A.-H., Khandekar M., Virtanen K.A., Nuutila P., Schaart G. (2012). Beige Adipocytes Are a Distinct Type of Thermogenic Fat Cell in Mouse and Human. Cell.

[12] Scherer P.E., Williams S., Fogliano M., Baldini G., Lodish H.F. (1995). A Novel Serum Protein Similar to C1q, Produced Exclusively in Adipocytes. J. Biol. Chem..

[13] Hu E., Liang P., Spiegelman B.M. (1996). AdipoQ Is a Novel Adipose-Specific Gene Dysregulated in Obesity. J. Biol. Chem..

[14] Wong G.W., Wang J., Hug C., Tsao T.-S., Lodish H.F. (2004). A Family of Acrp30/Adiponectin Structural and Functional Paralogs. Proc. Natl. Acad. Sci. USA.

[15] Schraw T., Wang Z.V., Halberg N., Hawkins M., Scherer P.E. (2008). Plasma Adiponectin Complexes Have Distinct Biochemical Characteristics. Endocrinology.

[16] Ruan H., Dong L.Q. (2016). Adiponectin Signaling and Function in Insulin Target Tissues. J. Mol. Cell Biol..

[17] Yamauchi T., Kamon J., Ito Y., Tsuchida A., Yokomizo T., Kita S., Sugiyama T., Miyagishi M., Hara K., Tsunoda M. (2003). Cloning of Adiponectin Receptors That Mediate Antidiabetic Metabolic Effects. Nature.

[18] Ding S.T., Liu B.H., Ko Y.H. (2004). Cloning and Expression of Porcine Adiponectin and Adiponectin Receptor 1 and 2 Genes in Pigs. J. Anim. Sci..

[19] Yamauchi T., Kadowaki T. (2013). Adiponectin Receptor as a Key Player in Healthy Longevity and Obesity-Related Diseases. Cell Metab..

[20] Liu B.-H., Lin Y.-Y., Wang Y.-C., Huang C.-W., Chen C.-C., Wu S.-C., Mersmann H.J., Cheng W.T.K., Ding S.-T. (2013). Porcine Adiponectin Receptor 1 Transgene Resists High-Fat/Sucrose Diet-Induced Weight Gain, Hepatosteatosis and Insulin Resistance in Mice. Exp. Anim..

[21] Lin Y.Y., Chen C.Y., Lin Y., Chiu Y.P., Chen C.C., Liu B.H., Mersmann H.J., Wu S.C., Ding S.T. (2013). Modulation of Glucose and Lipid Metabolism by Porcine Adiponectin Receptor 1–Transgenic Mesenchymal Stromal Cells in Diet-Induced Obese Mice. Cytotherapy.

[22] Lin Y.Y., Chen C.Y., Chuang T.Y., Lin Y., Liu H.Y., Mersmann H.J., Wu S.C., Ding S.T. (2014). Adiponectin Receptor 1 Regulates Bone Formation and Osteoblast Differentiation by GSK-3β/β-Catenin Signaling in Mice. Bone.

[23] Lin Y.-Y., Chen C.-Y., Ding S.-T. (2017). Adiponectin Receptor 1 Resists the Decline of Serum Osteocalcin and GPRC6A Expression in Ovariectomized Mice. PLoS ONE.

[24] Peng Y.-J., Shen T.-L., Chen Y.-S., Mersmann H.J., Liu B.-H., Ding S.-T. (2018). Adiponectin and Adiponectin Receptor 1 Overexpression Enhance Inflammatory Bowel Disease. J. Biomed. Sci..

[25] Zhang F., Hao G., Shao M., Nham K., An Y., Wang Q., Zhu Y., Kusminski C.M., Hassan G., Gupta R.K. (2018). An Adipose Tissue Atlas: An Image-Guided Identification of Human-like BAT and Beige Depots in Rodents. Cell Metab..

[26] Seale P., Kajimura S., Yang W., Chin S., Rohas L.M., Uldry M., Tavernier G., Langin D., Spiegelman B.M. (2007). Transcriptional Control of Brown Fat Determination by PRDM16. Cell Metab..

[27] Rosenwald M., Perdikari A., Rülicke T., Wolfrum C. (2013). Bi-Directional Interconversion of Brite and White Adipocytes. Nat. Cell Biol..

[28] Fischer B., Lassen U., Mortensen J., Larsen S., Loft A., Bertelsen A., Ravn J., Clementsen P., Høgholm A., Larsen K. (2009). Preoperative Staging of Lung Cancer with Combined PET–CT. N. Engl. J. Med..

[29] Ben-Haim S., Ell P. (2009). 18F-FDG PET and PET/CT in the Evaluation of Cancer Treatment Response. J. Nucl. Med..

[30] Bar-Shalom R., Yefremov N., Guralnik L., Gaitini D., Frenkel A., Kuten A., Altman H., Keidar Z., Israel O. (2003). Clinical Performance of PET/CT in Evaluation of Cancer: Additional Value for Diagnostic Imaging and Patient Management. J. Nucl. Med..

[31] Farwell M.D., Pryma D.A., Mankoff D.A. (2014). PET/CT Imaging in Cancer: Current Applications and Future Directions. Cancer.

[32] Kim S.-N., Jung Y.-S., Kwon H.-J., Seong J.K., Granneman J.G., Lee Y.-H. (2016). Sex Differences in Sympathetic Innervation and Browning of White Adipose Tissue of Mice. Biol. Sex Differ..

[33] Martínez de Morentin P.B., González-García I., Martins L., Lage R., Fernández-Mallo D., Martínez-Sánchez N., Ruíz-Pino F., Liu J., Morgan D.A., Pinilla L. (2014). Estradiol Regulates Brown Adipose Tissue Thermogenesis via Hypothalamic AMPK. Cell Metab..

[34] Grefhorst A., Van den Beukel J.C., Van Houten E.L.A., Steenbergen J., Visser J.A., Themmen A.P. (2015). Estrogens Increase Expression of Bone Morphogenetic Protein 8b in Brown Adipose Tissue of Mice. Biol. Sex Differ..

[35] Kajimura S., Spiegelman B.M., Seale P. (2015). Brown and Beige Fat: Physiological Roles beyond Heat Generation. Cell Metab..

[36] Bartelt A., Bruns O.T., Reimer R., Hohenberg H., Ittrich H., Peldschus K., Kaul M.G., Tromsdorf U.I., Weller H., Waurisch C. (2011). Brown Adipose Tissue Activity Controls Triglyceride Clearance. Nat. Med..

[37] Cypess A.M., Weiner L.S., Roberts-Toler C., Elía E.F., Kessler S.H., Kahn P.A., English J., Chatman K., Trauger S.A., Doria A. (2015). Activation of Human Brown Adipose Tissue by a Β3-Adrenergic Receptor Agonist. Cell Metab..

[38] Yoneshiro T., Aita S., Matsushita M., Kayahara T., Kameya T., Kawai Y., Iwanaga T., Saito M. (2013). Recruited Brown Adipose Tissue as an Antiobesity Agent in Humans. J. Clin. Investig..

[39] Van der Lans A.A.J.J., Hoeks J., Brans B., Vijgen G.H.E.J., Visser M.G.W., Vosselman M.J., Hansen J., Jörgensen J.A., Wu J., Mottaghy F.M. (2013). Cold Acclimation Recruits Human Brown Fat and Increases Nonshivering Thermogenesis. J. Clin. Investig..

[40] Ouellet V., Labbé S.M., Blondin D.P., Phoenix S., Guérin B., Haman F., Turcotte E.E., Richard D., Carpentier A.C. (2012). Brown Adipose Tissue Oxidative Metabolism Contributes to Energy Expenditure during Acute Cold Exposure in Humans. J. Clin. Investig..

[41] Blondin D.P., Labbé S.M., Tingelstad H.C., Noll C., Kunach M., Phoenix S., Guérin B., Turcotte É.E., Carpentier A.C., Richard D. (2014). Increased Brown Adipose Tissue Oxidative Capacity in Cold-Acclimated Humans. J. Clin. Endocrinol. Metab..

[42] Hanssen M.J.W., Van der Lans A.A.J.J., Brans B., Hoeks J., Jardon K.M.C., Schaart G., Mottaghy F.M., Schrauwen P., Van Marken Lichtenbelt W.D. (2016). Short-Term Cold Acclimation Recruits Brown Adipose Tissue in Obese Humans. Diabetes.

[43] Lim S., Honek J., Xue Y., Seki T., Cao Z., Andersson P., Yang X., Hosaka K., Cao Y. (2012). Cold-Induced Activation of Brown Adipose Tissue and Adipose Angiogenesis in Mice. Nat. Protoc..

[44] Kim J.-Y., Van de Wall E., Laplante M., Azzara A., Trujillo M.E., Hofmann S.M., Schraw T., Durand J.L., Li H., Li G. (2007). Obesity-Associated Improvements in Metabolic Profile through Expansion of Adipose Tissue. J. Clin. Investig..

[45] Combs T.P., Pajvani U.B., Berg A.H., Lin Y., Jelicks L.A., Laplante M., Nawrocki A.R., Rajala M.W., Parlow A.F., Cheeseboro L. (2004). A Transgenic Mouse with a Deletion in the Collagenous Domain of Adiponectin Displays Elevated Circulating Adiponectin and Improved Insulin Sensitivity. Endocrinology.

[46] Kubota N., Yano W., Kubota T., Yamauchi T., Itoh S., Kumagai H., Kozono H., Takamoto I., Okamoto S., Shiuchi T. (2007). Adiponectin Stimulates AMP-Activated Protein Kinase in the Hypothalamus and Increases Food Intake. Cell Metab..

[47] Saito K., Arata S., Hosono T., Sano Y., Takahashi K., Choi-Miura N.-H., Nakano Y., Tobe T., Tomita M. (2006). Adiponectin Plays an Important Role in Efficient Energy Usage under Energy Shortage. Biochim. Biophys. Acta BBA Mol. Cell Biol. Lipids.

[48] Kajimura D., Lee H.W., Riley K.J., Arteaga-Solis E., Ferron M., Zhou B., Clarke C.J., Hannun Y.A., DePinho R.A., Guo X.E. (2013). Adiponectin Regulates Bone Mass via Opposite Central and Peripheral Mechanisms through FoxO1. Cell Metab..

[49] Fruebis J., Tsao T.-S., Javorschi S., Ebbets-Reed D., Erickson M.R.S., Yen F.T., Bihain B.E., Lodish H.F. (2001). Proteolytic Cleavage Product of 30-KDa Adipocyte Complement-Related Protein Increases Fatty Acid Oxidation in Muscle and Causes Weight Loss in Mice. Proc. Natl. Acad. Sci. USA.

[50] Zhou L., Deepa S.S., Etzler J.C., Ryu J., Mao X., Fang Q., Liu D.D., Torres J.M., Jia W., Lechleiter J.D. (2009). Adiponectin Activates AMP-Activated Protein Kinase in Muscle Cells via APPL1/LKB1-Dependent and Phospholipase C/Ca2+/Ca2+/Calmodulin-Dependent Protein Kinase Kinase-Dependent Pathways. J. Biol. Chem..

[51] Seale P., Conroe H.M., Estall J., Kajimura S., Frontini A., Ishibashi J., Cohen P., Cinti S., Spiegelman B.M. (2011). Prdm16 Determines the Thermogenic Program of Subcutaneous White Adipose Tissue in Mice. J. Clin. Investig..

[52] Chouchani E.T., Kajimura S. (2019). Metabolic Adaptation and Maladaptation in Adipose Tissue. Nat. Metab..

[53] Imai J., Katagiri H., Yamada T., Ishigaki Y., Ogihara T., Uno K., Hasegawa Y., Gao J., Ishihara H., Sasano H. (2006). Cold Exposure Suppresses Serum Adiponectin Levels through Sympathetic Nerve Activation in Mice. Obesity.

[54] Dong M., Yang X., Lim S., Cao Z., Honek J., Lu H., Zhang C., Seki T., Hosaka K., Wahlberg E. (2013). Cold Exposure Promotes Atherosclerotic Plaque Growth and Instability via UCP1-Dependent Lipolysis. Cell Metab..

[55] Qiao L., Sun Yoo H., Bosco C., Lee B., Feng G.-S., Schaack J., Chi N.-W., Shao J. (2014). Adiponectin Reduces Thermogenesis by Inhibiting Brown Adipose Tissue Activation in Mice. Diabetologia.

[56] Zhu Q., Glazier B.J., Hinkel B.C., Cao J., Liu L., Liang C., Shi H. (2019). Neuroendocrine Regulation of Energy Metabolism Involving Different Types of Adipose Tissues. Int. J. Mol. Sci..

[57] Qiao L., Kinney B., Schaack J., Shao J. (2011). Adiponectin Inhibits Lipolysis in Mouse Adipocytes. Diabetes.

[58] Wang L., Luo Y., Luo L., Wu D., Ding X., Zheng H., Wu H., Liu B., Yang X., Silva F. (2021). Adiponectin Restrains ILC2 Activation by AMPK-Mediated Feedback Inhibition of IL-33 Signaling. J. Exp. Med..

[59] Chen Y.-J., Liu H.-Y., Chang Y.-T., Cheng Y.-H., Mersmann H.J., Kuo W.-H., Ding S.-T. (2016). Isolation and Differentiation of Adipose-Derived Stem Cells from Porcine Subcutaneous Adipose Tissues. JoVE J. Vis. Exp..

[60] Huang C.-W., Chen Y.-J., Yang J.-T., Chen C.-Y., Ajuwon K.M., Chen S.-E., Su N.-W., Chen Y.-S., Mersmann H.J., Ding S.-T. (2017). Docosahexaenoic Acid Increases Accumulation of Adipocyte Triacylglycerol through Up-Regulation of Lipogenic Gene Expression in Pigs. Lipids Health Dis..

[61] Huang C.-W., Chien Y.-S., Chen Y.-J., Ajuwon K.M., Mersmann H.M., Ding S.-T. (2016). Role of N-3 Polyunsaturated Fatty Acids in Ameliorating the Obesity-Induced Metabolic Syndrome in Animal Models and Humans. Int. J. Mol. Sci..

