# Supplementary figures and images for: Overexpression of Adiponectin Receptor 1 Inhibits Brown and Beige Adipose Tissue Activity in Mice
Source: Int J Mol Sci. 2021 Jan 18;22(2):906. doi: 10.3390/ijms22020906 (PMC7831094; doi:10.3390/ijms22020906)

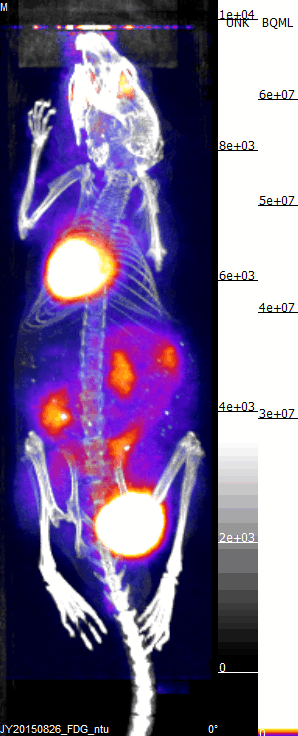

Supplement: Supplementary file 1 [file ijms-22-00906-s001.zip › Supplemental Tables and Videos /Supplemental Materials-Videos/Male/AdipoR1-2 Male 3D.gif]

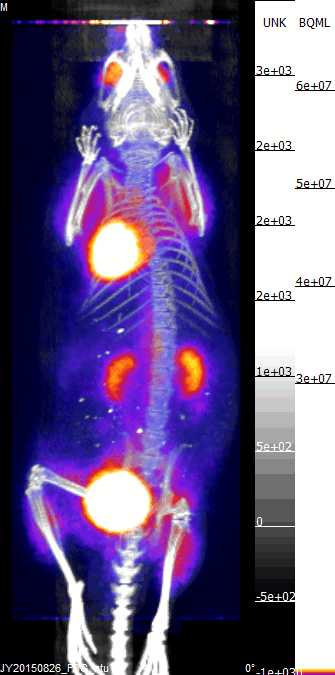

Supplement: Supplementary file 1 [file ijms-22-00906-s001.zip › Supplemental Tables and Videos /Supplemental Materials-Videos/Male/Wid-type Male 3D.gif]

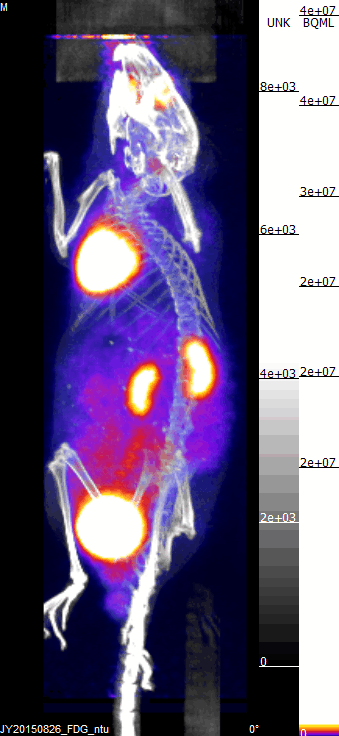

Supplement: Supplementary file 1 [file ijms-22-00906-s001.zip › Supplemental Tables and Videos /Supplemental Materials-Videos/Female/AdipoR1 Female 3D.gif]

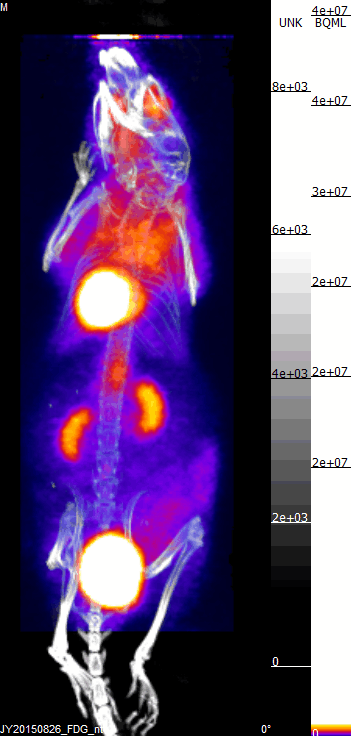

Supplement: Supplementary file 1 [file ijms-22-00906-s001.zip › Supplemental Tables and Videos /Supplemental Materials-Videos/Female/Wild-type Female 3D.gif]
